# C-Terminal Engineering of CXCL12 and CCL5 Chemokines: Functional Characterization by Electrophysiological Recordings

**DOI:** 10.1371/journal.pone.0087394

**Published:** 2014-01-31

**Authors:** Antoine Picciocchi, Lina Šiaučiūnaiteė-Gaubard, Isabelle Petit-Hartlein, Rabia Sadir, Jean Revilloud, Lydia Caro, Michel Vivaudou, Franck Fieschi, Christophe Moreau, Corinne Vivès

**Affiliations:** 1 Université Grenoble Alpes, Institut de Biologie Structurale, Grenoble, France; 2 CEA, DSV/IBS, Grenoble, France; 3 CNRS, IBS, Grenoble, France; 4 Institut Universitaire de France, Paris, France; University of Leuven, Rega Institute, Belgium

## Abstract

Chemokines are chemotactic cytokines comprised of 70–100 amino acids. The chemokines CXCL12 and CCL5 are the endogenous ligands of the CXCR4 and CCR5 G protein-coupled receptors that are also HIV co-receptors. Biochemical, structural and functional studies of receptors are ligand-consuming and the cost of commercial chemokines hinders their use in such studies. Here, we describe methods for the expression, refolding, purification, and functional characterization of CXCL12 and CCL5 constructs incorporating C-terminal epitope tags. The model tags used were hexahistidines and Strep-Tag for affinity purification, and the double lanthanoid binding tag for fluorescence imaging and crystal structure resolution. The ability of modified and purified chemokines to bind and activate CXCR4 and CCR5 receptors was tested in *Xenopus* oocytes expressing the receptors, together with a Kir3 G-protein activated K^+^ channel that served as a reporter of receptor activation. Results demonstrate that tags greatly influence the biochemical properties of the recombinant chemokines. Besides, despite the absence of any evidence for CXCL12 or CCL5 C-terminus involvement in receptor binding and activation, we demonstrated unpredictable effects of tag insertion on the ligand apparent affinity and efficacy or on the ligand dissociation. These tagged chemokines should constitute useful tools for the selective purification of properly-folded chemokines receptors and the study of their native quaternary structures.

## Introduction

G protein-coupled receptors (GPCRs) are membrane proteins crucial for physiological functions and environment sensing. They represent major pharmaceutical targets with over 30% of currently-marketed drugs acting on GPCRs [1]. Among GPCRs, the chemokine receptors CXCR4 and CCR5 have gained attention as they are hijacked by HIV virus to serve as entry points into cells. The natural ligands of CXCR4 and CCR5 are small proteins of 70–100 residues such as the CXCL12 and CCL5 chemokines. Those chemokines are defined by very specific folds constrained by highly conserved disulfide bridges. Low quantities of chemokines are affordable *via* biochemical suppliers. However, structural studies of chemokine receptors based on affinity selection of functional purified receptors require a large amount of immobilized and functional chemokines. The length and the fold of the chemokines make their chemical synthesis complex and expensive, therefore we used an alternative low-cost approach based on bacterial production in inclusion bodies. While most protocols for CXCL12 and CCL5 production described a refolding procedure from inclusion bodies [2], one study presented a functional expression mode through a fusion strategy with the maltose binding protein [3]. We tested here both approaches to produce CXCL12 and CCL5 chemokines incorporating representative tags for affinity selection and biophysical studies of their cognate receptors. Selected affinity tags were hexahistidines for ion metal affinity chromatography (IMAC), and Strep-Tag for StrepTactin-affinity chromatography or anchorage on StrepTactin-coated surfaces of surface plasmon resonance sensor chips. For applications in fluorescence imaging or phasing in crystallography, we selected the double lanthanoid binding tag (LT). This tag is a 35-residue sequence that selectively binds lanthanoid ions and then emits fluorescent light upon UV stimulation [4]. Alterations of N-terminal domains of chemokines dramatically impede their functionality, including their potency as HIV inhibitors [5]–[8]. We therefore appended the tags to the C-terminus of the proteins. To create multi-functional chemokines, we also combined affinity tags and LT-tag in the same chemokine construct.

While the C-terminus of chemokines does not seem critical for their function, insertion of one or several tags could affect their binding and efficacy on the receptor. Several functional assays are available to test receptor binding and signaling: radioactive or fluorescent labeled ligands, [9] and downstream tests that monitor various points of the receptor-activated signaling cascade, from agonist-stimulated radioactive GTP binding to G proteins [10], cytosolic Ca^2+^ variations, changes in cAMP concentration to cell morphological changes [11] or receptor translocation [12].

**Figure 1:**
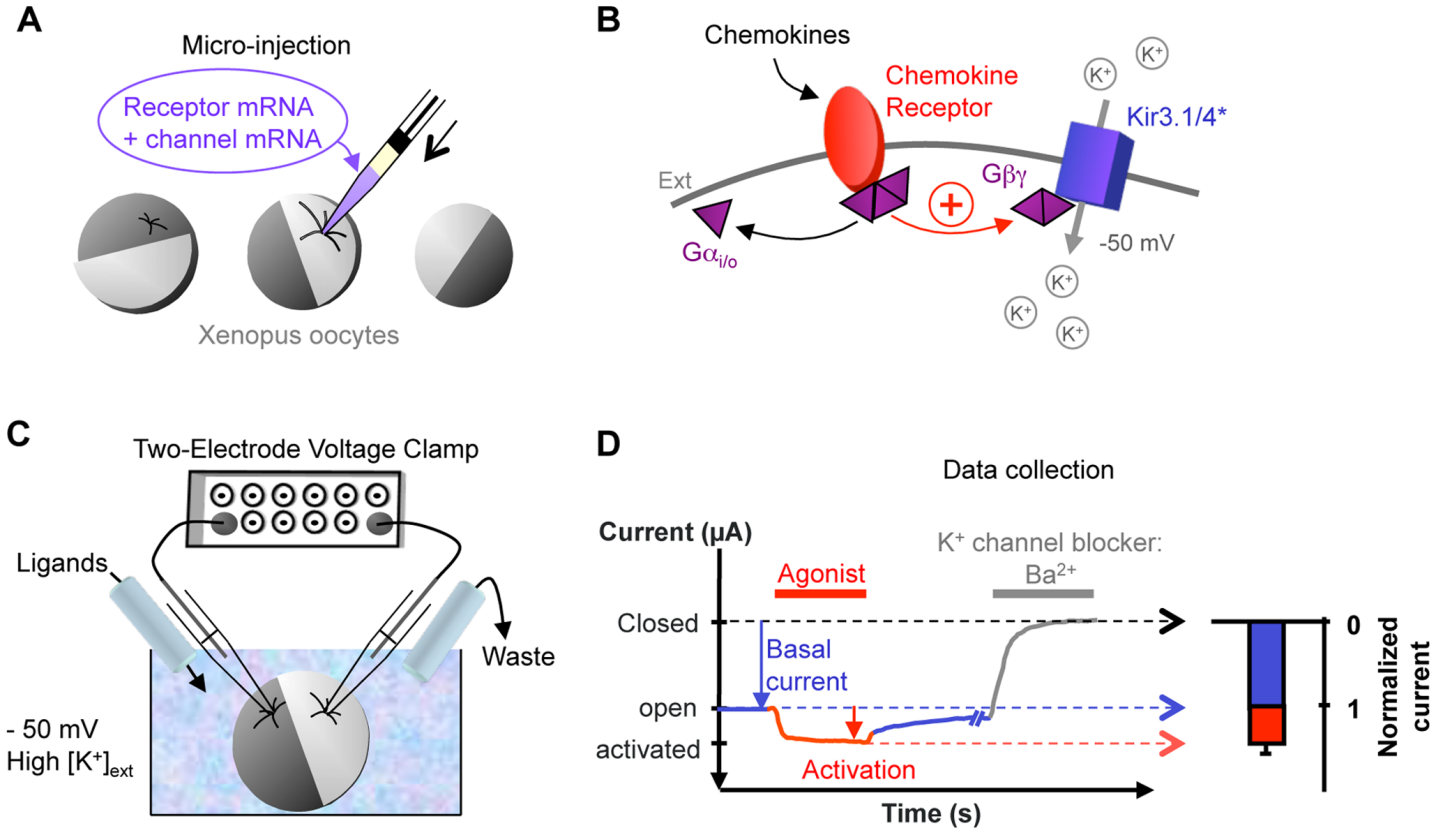
Principle of the electrophysiological characterization of chemokine receptors. A. Receptors and channels are expressed in *Xenopus laevis* oocytes by mRNA micro-injection. After 2 day-incubation, purified chemokines are electrophysiologically characterized. B. Schematic representation of a *Xenopus* oocyte plasma membrane containing a heterologously-expressed chemokine receptor and the G protein-activated Kir3.1* or Kir3.4* channels. The Kir3.x* channel is mutated to function as a homomeric channel. Binding of chemokines to the receptor induces activation and release of the G protein subunits. Gβγ subunits activate the Kir3.1* channels by direct binding, resulting in an increase in ion current carried by K^+^. C. Schematic representation of the Two-Electrode Voltage Clamp set-up. The oocyte is impaled by 2 glass pipettes containing 3 M KCl and an Ag/AgCl electrode. Electrical current recording is performed under continuous flow of buffer +/− ligands or channel blockers. Change of solutions is controlled by a semi-automatic perfusion system. The used TEVC bath has a potassium concentration similar to the intracellular K^+^ concentration. D. Representative TEVC recording of Kir3* generated current at −50 mV. The basal current generated by the channels in basal state is determined in the first minute and represented in blue line and bar. This basal current is the reference (100%) for the normalization of ligand-induced effect represented in red line and bar. Barium (Ba^2+^) is a generic blocker of K^+^ channels and positions the barium-sensitive baseline.

To assess the consequences of chemokine modifications both on receptor binding capacity and signal transduction, we used here a simple functional assay based on co-expression of chemokine receptors and G protein-activated channels (GIRK or Kir3 channels) in *Xenopus* oocytes and Two-Electrode Voltage-Clamp recordings (Figure 1). This method allowed us to record in real-time the receptor activation and dose-response curves were easily executed by sequential applications of increased ligand concentrations in constant flow. With this approach we could determine on single cells the effect of the tag insertions on the apparent receptor affinity, the binding efficacy and the receptor de-activation.

This technique is particularly appropriate for the functional characterization of external ligands, such as the chemokines, and does require neither purified receptors nor radiolabeling. The sensitivity is amplified by the activation of multiple G proteins by the same receptor and the signal is correlated with the ligand concentration.

This article describes the expression and purification of various chemokine constructs designed as new tools to carry out ligand or chemokine receptor studies. The effects of different tags in different combinations have been investigated on the expression, purification and activity of CXCL12 and CCL5 chemokines. The unpredictable nature of the herein reported effects strengthens the necessity to characterize the addition of any new tags to the chemokine proteins.

## Materials and Methods

### Engineering of Expression Vectors

**Figure 2:**
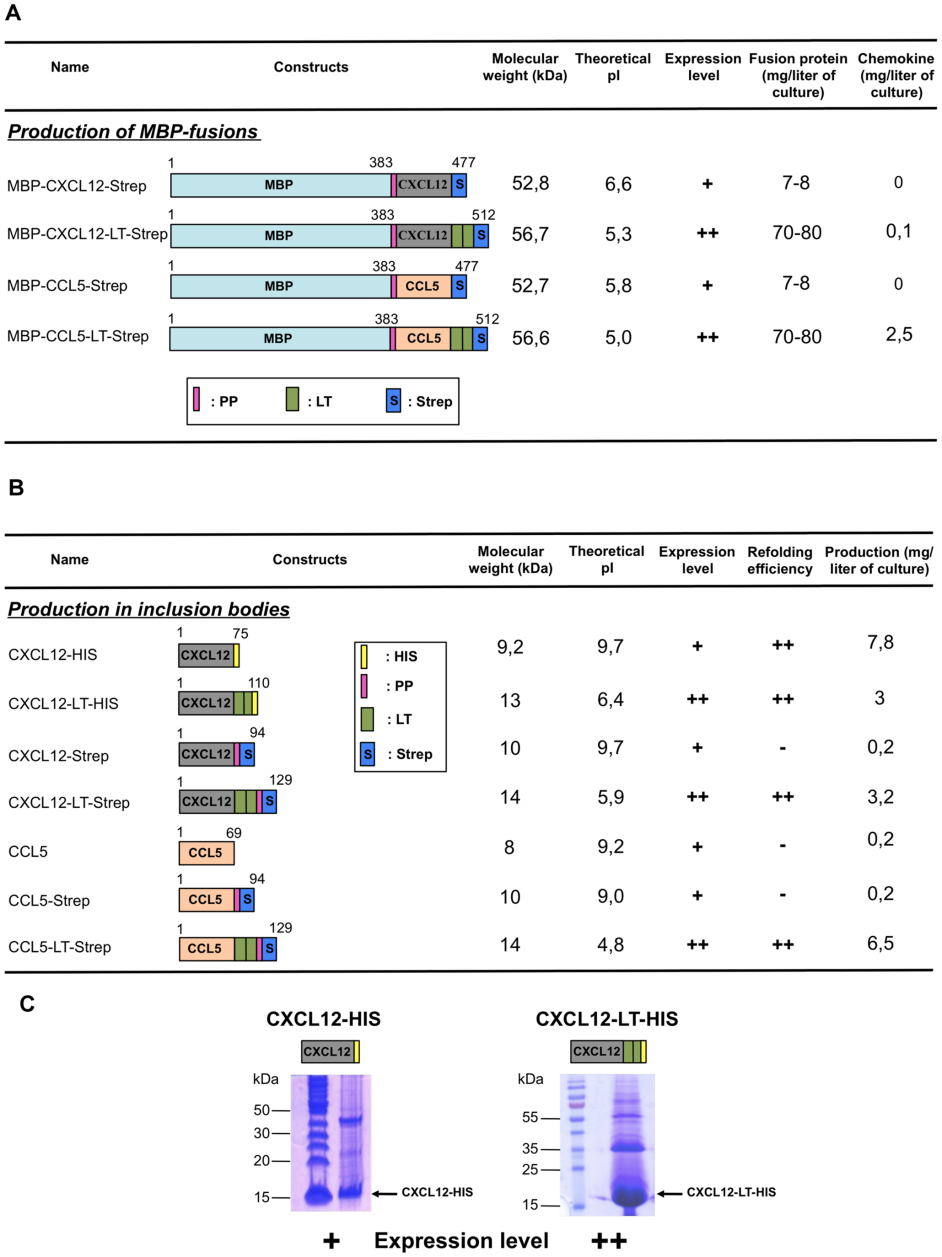
Scheme of expression pattern and predicted properties of the different chemokines used in this work. A. MBP-chemokine constructs. B. Chemokines expressed in *E. coli* inclusion bodies. Names and sequences of each construct are shown on the left. Numbers represent amino acid positions. The theoretical molecular weight and isoelectric point were determined with the Expasy ProtParam tool (http://web.expasy.org/protparam/). Chemokine expression level and purification yield are indicated. The highest chemokine levels are highlighted in bold. MBP: Maltose Binding Protein; PP: Prescission protease site; S: Strep-tag; HIS: HIS-tag; LT: Lanthanoid binding tag; (+): good expression level; (++): strong expression level. C. Purified inclusion bodies (15 µL per lane) prepared from *E. coli* cells over-expressing CXCL12-HIS and CXCL12-LT-HIS proteins were analysed by SDS/PAGE and stained with a Coomassie solution. PageRuler Unstained Protein Ladder and PageRuler PreStained Protein Ladder were used for CXCL12-HIS and CXCL12-LT-HIS gels, respectively.

Standard pUC57 plasmids containing optimized synthetic human genes designed for the efficient production of chemokines in *E. coli* were manufactured by GeneCust (Evry, France). After PCR amplifications using suitable primers, cDNAs encoding the full-length chemokines were subcloned into pET20-b (Novagen) (chemokines expression in *E. coli* inclusion bodies) or pMAL-C4x (New England Biolabs) (chemokines expression with a N-terminal fused maltose binding protein) expression plasmids (Figure 2). The various constructs displayed a C-terminal HIS or Strep-tag (Strep). In some cases, a Pre-Scission protease site (PP) was added for cleavage and double-lanthanoid-binding tags (LT) [4] were inserted right after the C-terminus of the proteins, before other tags and cleavage sites. All constructs were confirmed by sequencing. Amino acid sequences were: HIS = HHHHHH; Strep: WSHPQFEK; PP = LEVLFQGP; LT = GPGYIDTNNDGWIEGDELYIDTNNDGWIEGDELLA.

### Expression of Recombinant Chemokines

WT CXCL12 was chemically synthetized by F. Baleux, Pasteur Institute, Paris and the WT CCL5 was purchased from Immunotools. The molecular weight markers (PageRuler Unstained and PreStained Protein Ladder) were purchased from Fermentas.

*E. coli* BL21 (DE3) cells transformed with the different expression plasmids were grown at 37°C in Luria Bertani medium supplemented with 100 µg.mL^−1^ ampicillin until cultures reached an optical density of 0.6 to 0.8 at 600 nm. Then, 1 mM isopropylthio-β-D-galactopyranoside was added to the cultures in order to induce expression of recombinant proteins. Cells were further incubated for 3 h at 37°C (CXCL12-HIS, CXCL12-Strep and CCL5-Strep) or 16 h at 20°C (MBP-CXCL12-Strep, MBP-CCL5-Strep, CXCL12-LT-HIS, CXCL12-LT-Strep and CCL5-LT-Strep) and harvested by centrifugation for 30 min at 5,000 g.

### MBP-CXCL12-Strep and MBP-CCL5-Strep Purification

Bacterial cells expressing the MBP-fused chemokines were resuspended in buffer A (100 mM Tris-HCl pH 8.0, 150 mM NaCl, 1 mM EDTA) containing Complete protease inhibitors (Roche) and disrupted twice using a Microfluidizer M-110P (Microfluidics international, Newton, MA) at 10,000 psi. After centrifugation for 45 min at 20,000 g, soluble MBP-chemokines present in the supernatant were purified by affinity chromatography using a StrepTactin column (5 mL, Amersham Biosciences) previously equilibrated in buffer A. After an extensive washing step, recombinant chemokines were eluted with buffer A supplemented with 2.5 mM of desthiobiotin. After purification, the N-terminal MBP-domain of the fusion proteins was cleaved off after overnight incubation at room temperature with Pre-Scission protease (1.75 U protease/mg of fusion proteins). Removal of the MBP moiety was performed by purification over an amylose column (BioLabs). The unbound fraction that contained the chemokine was concentrated and run over a Superdex 200 gel filtration column.

### Inclusion Bodies Preparation

Bacterial cells expressing chemokines in inclusion bodies were resuspended in buffer B (50 mM Tris-HCl, pH 8.0) supplemented with Complete protease inhibitors (Roche). Bacterial pellets were disrupted as described above. After centrifugation for 30 min at 20,000 g, inclusion bodies were washed with buffer B supplemented with 2 M Urea and 5% Triton X100, then with buffer B containing 2 M Urea and finally with buffer B alone.

### CXCL12-HIS and CXCL12-Strep Refolding and Purification

Inclusion bodies were solubilized for 20 min at 50°C in buffer B with 7.5 M Guanidine hydrochloride (GdnHCl), 5 mM EDTA and 20 mM DTT. Refolding was performed by rapid dilution with buffer B down to 1 M GdnHCl. The mixture was gently stirred overnight at 4°C after addition of Complete protease inhibitors (Roche), diluted 4 times with buffer B and loaded onto a Capto S column (5 mL, Amersham Biosciences) equilibrated with 50 mM Tris-HCl, pH 7.5. Chemokines were then eluted with a NaCl gradient (0–1 M), concentrated using Centricon centrifugal concentrator device (Amicon) and further purified over a Superdex 200 gel filtration column (Amersham Biosciences) previously equilibrated with 20 mM Na_2_HPO_4_, pH 6.0. Purified proteins were analyzed by ion-spray mass spectrometry and stored at –80°C until use.

### CCL5 and CCL5-Strep Refolding and Purification

Inclusion bodies were solubilized for 30 min at 60°C in buffer B with 6 M GdnHCl and 1 mM DTT. After centrifugation for 30 min at 20,000 g, the clear supernatant was dialyzed 4 hours at room temperature against 1% acetic acid in order to precipitate impurities. The concentrated, clear supernatant was diluted down to 1 mg/mL of protein and refolding was achieved by drop-wise dilution in the adequate refolding buffer (20 mM Tris-HCl pH 8.0, 0.01 mM oxidized glutathione and 0.1 mM reduced glutathione). After an overnight incubation at 4°C with mild stirring, the solution was adjusted to pH 4.5 and subsequently loaded onto a Capto S column (5 mL, Amersham Biosciences) pre-equilibrated with 50 mM Na-Acetate, pH 4.5. The protein was then eluted with a NaCl gradient (0–1 M), concentrated with Centricon cell and further purified over a Superdex 200 gel filtration column previously equilibrated with 20 mM Na_2_HPO_4_, pH 6.0, 150 mM NaCl.

### CXCL12-LT-HIS, CXCL12-LT-Strep and CCL5-LT-Strep Refolding and Purification

Inclusion bodies were solubilized for 1 h at room temperature in buffer C (100 mM Tris-HCl, pH 8.0) supplemented with 6 M Urea, 100 mM NaCl, 0.5 mM EDTA and 5 mM DTT. Refolding was achieved by drop-wise dilution into a volume 100 times that of the Urea solution of buffer C containing 0.5 mM EDTA, 0.2 mM oxidized glutathione and 1 mM reduced glutathione. The respective refolding solution was stirred overnight at 4°C and loaded onto a 10 mL Q-sepharose column (Amersham Biosciences) pre-equilibrated in buffer B. A linear gradient elution was performed over 100 mL from 0 to 1 M NaCl in buffer B and the fractions containing the refolded chemokines were concentrated with Centricon cell and further purified over a Superdex 200 gel filtration column equilibrated with buffer B containing 150 mM NaCl.

### Protein Quantification and Spectrometric Characterization

Protein concentration was determined by measuring the absorbance at 280 nm and using the following theoretical molar extinction coefficients determined with the Expasy ProtParam tool (http://web.expasy.org/protparam/): MBP-CXCL12-Strep (80580 M^−1^.cm^−1^), MBP-CXCL12-LT-Strep (94560 M^−1^.cm^−1^), MBP-CCL5-Strep (85050 M^−1^.cm^−1^), MBP-CCL5-LT-Strep (99030 M^−1^.cm^−1^), CXCL12-HIS (8730 M^−1^.cm^−1^), CXCL12-LT-HIS (22710 M^−1^.cm^−1^), CXCL12-Strep (14230 M^−1^.cm^−1^), CXCL12-LT-Strep (28210 M^−1^.cm^−1^), CCL5 (13200 M^−1^.cm^−1^), CCL5-Strep (18700 M^−1^.cm^−1^), CCL5-LT-Strep (32680 M^−1^.cm^−1^). CXCL12-HIS (20 µM in 20 mM sodium phosphate, pH 6.0) and CXCL12-LT-HIS (20 µM in 20 mM sodium phosphate, pH 8.0) circular dichroism (CD) spectra were measured at 25°C in a Jobin Yvon CD6 spectropolarimeter over a 190- to 260-nm range (1-nm interval) in a 0,1 cm optical path length quartz cuvette. Three repetitive scans were taken for each sample and corrected for solvent contributions. Molar ellipticity was calculated as previously described [13]. Protein masses were determined using an electrospray TOF mass spectrometer (Agilent, LC/MSD TOF) directly coupled with the HPLC system (Agilent 1100 series).

### Terbium Titration and SDS-PAGE Analysis

Titrations were recorded with a Photon Technology International Quanta Master I fluorimeter using 1 cm path length quartz cuvette and slit widths of 2 and 4 nm. Tryptophan-sensitized Tb^3+^ luminescence was collected at room temperature by exciting the sample at 280 nm. The purified CXCL12-LT-HIS protein (2 mL at 75 µM in Hepes 20 mM pH 8.0; 100 mM NaCl) was titrated by adding 2 µL aliquots of 15 mM Tb^3+^. After each addition, the solution was mixed and the luminescence emission spectrum was recorded between 450 and 550 nm.

For SDS-PAGE analysis, proteins were loaded onto two 15% polyacrylamide gels in denaturing buffer and subjected to electrophoresis at 220 V for 1 hour. After migration, one gel was immediately stained with a Coomassie solution whereas the other one was washed twice for 20 minutes in buffer D (100 mM NaCl, 10 mM Hepes, pH 7.0), followed by a 1 hour incubation in buffer C containing 50 µM of TbCl_3_. Luminescent bands were visualized on a UV-transilluminator (Gel Doc 2000, Bio-Rad).

### Co-expression of Receptors and Channels in *Xenopus* Oocytes

Human CXCR4 and CCR5 genes were subcloned into a pGH vector designed for protein overexpression in *Xenopus* oocyte [14]. The Kir3.1_F137S_ (Kir3.1*) and Kir3.4_S143T_ (Kir3.4*) genes were inserted in the same vector. Kir3.1 and Kir3.4 form heterotetramers physiologically and are activated by Gi/o heterotrimeric proteins. The mutations F137S in Kir3.1 and S143T in Kir3.4 allow the formation of functional homotetramers simplifying the system to one subunit [15]. The two constructs are interchangeable and give the same response. Both channels were used. After linearization of the plasmids, mRNAs were synthesized *in vitro* using the T7 mMessage mMachine Kit (Invitrogen). mRNAs were purified by standard phenol:chloroform extraction protocol, analyzed on agarose gel and quantified by spectrophotometry.

Animal handling and experiments fully conformed with French regulations and were approved by local governmental veterinary services (authorization no. 38-08-10 from the Ministère de l’Agriculture, Direction des Services Vétérinaires to Michel Vivaudou). Oocytes were surgically removed from *Xenopus laevis* and defolliculated by 2 hour incubations in 2 mg.mL^−1^ type 1A collagenase solution at 19°C. Stage V and VI oocytes were microinjected with 50 nL of RNase-free water containing a mixture of the following quantities of RNA: CXCR4 or CCR5, 2.5 ng; Kir3.1* or Kir3.4*, 2.5 ng. Microinjected oocytes were incubated for >2 days at 19°C in Barth’s solution (1 mM KCl, 0.82 mM MgSO_4_, 88 mM NaCl, 2.4 mM NaHCO_3_, 0.41 mM CaCl_2_, 16 mM Hepes, pH 7.4) supplemented with 100 U.mL^−1^ penicillin, streptomycin and gentamycin. All chemicals were purchased from Sigma-Aldrich.

### Electrophysiological Recordings

Whole-cell currents were recorded with the two-electrode voltage clamp (TEVC) technique using a GeneClamp 500 amplifier (Molecular Devices). Microelectrodes were filled with 3 M KCl and oocytes were bathed in the following solution: 91 mM KCl, 1.8 mM CaCl_2_, 1 mM MgCl_2_, 5 mM HEPES, 0.3 mM niflumic acid (to block endogenous Cl^−^ currents), pH 7.4. The TEVC voltage protocol consisted of sequences of 500 ms steps to −50, 0 and +50 mV: during which current was measured: repeated every 5 s. The holding potential was 0 mV. The values shown in the figures are those recorded at −50 mV. In our TEVC configuration, solutions were applied in a constant flow over the impaled oocytes and the current generated by Kir3* channels was recorded in real-time. By convention, the recorded current is negative in our experimental conditions, and the first seconds of recordings (Figure 1D, blue arrow) correspond to the basal current generated by the channel in the control (in absence of chemokines). Application of agonists such as chemokines increases the negative current amplitude (Figure 1D, red arrow) and is normalized using the basal current as a reference (Figure 1D, red bar in the chart).

### Data Analysis

Basal current was measured while oocytes were in standard bath solution during the first minute of recording. Ba^2+^ (3 mM) was used as a generic potassium-channel blocker to establish the amount of exogenous current, designated as Ba^2+^-sensitive current. All values of current reported here refer to Ba^2+^-sensitive currents and were calculated by subtracting the current in Ba^2+^ from the values measured in each condition. For current normalization, changes in Ba^2+^-sensitive currents by effectors were calculated with respect to the value measured before application. Arrows in the figures indicate the time at which currents were measured. Concentration-response data were obtained by sequential application of increasing agonist concentrations, and changes in current were calculated only with respect to the current before application of the initial lowest concentration.

Average values are presented as mean±s.e.m. Non-linear least-square curve-fitting was carried out with Origin 8 software (OriginLab) using a standard Hill equation:
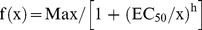
where x is the ligand concentration, Max the asymptotical maximal effect, EC_50_ the concentration for half-maximal effect, and h the Hill coefficient. The fits shown in the figures were performed using average data from several oocytes. For statistical analysis of parameters Max and EC_50_, individual dose-response data from each oocyte tested were fitted using the above equation with h = 1 to obtain a set of values of Max and EC_50_ for each construct and ligand. Statistical significance for these parameters and for other experimental data was established with unpaired two-tailed Student t-tests and is indicated as P-values in the text.

### Chemotaxis Assays

Migration of the lymphoblastic cell line Jurkat cells in response to CXCL12 or tagged-CXCL12 was evaluated using a transwell system. Briefly, cells were labelled with the intracellular fluorescent dye, calcein-AM (BD Biosciences), at 1 µM for 30 minutes at 37°C. Following labelling, cells were washed twice with PBS, and 2×10^5^ labelled cells in 100 µL of RPMI medium supplemented with 20 mM HEPES (*N*-2-hydroxyethylpiperazine-*N’*-2-ethanesulfonic acid) and 1% human AB serum were added to the upper chamber of a 96-well, 5-µm pore polycarbonate Transwell culture insert (Corning, Sigma). The same media (250 µL) containing 0 to 300 nM chemokine (either WT CXCL12, tagged-CXCL12, WT CCL5 or tagged-CCL5) was placed in the lower chamber. After 3 hours at 37°C in humidified air with 5% CO_2_, fluorescence of cells that had been chemoattracted through the filter to the lower wells was measured using a fluorescence plate reader (excitation filter 485 nm and emission filter 535 nm) (Wallac Victor, Perkin Elmer).

## Results and Discussion

### Expression and Purification of Chemokines

Different versions of the tagged chemokines CCL5 and CXCL12 were designed for affinity purification and biophysical studies of the CCR5 and CXCR4 receptors. All constructs are detailed in Figure 2.

### Soluble Expression of MBP Fusions

The first production attempt was based on the strategy adopted by Cho and co-workers [3] who reported high levels of soluble chemokine expression in *E. coli* when fused to a N-terminal Maltose Binding Protein MBP. Synthetic genes encoding CXCL12 and CCL5, optimized for *E.coli* expression, were designed to be cloned into the pMal 4x vectors. The final constructs MBP-CCL5-Strep and MBP-CXCL12–Strep displayed from N-term to C-term: the MBP sequence, a PreScission protease site to enable the subsequent cleavage of the fusion protein, the chemokine synthetic gene and a C-terminal Strep Tag. Similar constructs comprising an additional double lanthanoid binding tag (LT) were also designed: MBP-CCL5-LT-Strep and MBP-CXCL12-LT-Strep. This tag specifically binds Terbium (Tb) which fluorescent properties could be used to track the chemokine. All those constructs are represented in Figure 2A.

Protein expression was induced in *E. coli* BL21 (DE3) cells. After cell disruption, and removal of cellular debris, the supernatant was purified over a StrepTactin column. Although all MBP fusion proteins were expressed with satisfactory yields, a remarkable enhancement was provided by the addition of the LT tag. Yields of 7–8 mg.L^−1^ of culture were obtained for MBP-CCL5-Strep and MBP-CXCL12–Strep while the presence of the LT tag boosted the quantities up to 70–80 mg.L^−1^ of culture. However, the subsequent cleavage of the fusion protein led to the precipitation of the whole protein sample despite attempts to optimize the conditions. Once again the LT tag dramatically affected the protein behavior since its presence maintained the chemokine soluble after cleavage. Removal of the MBP moiety was performed by purification over an amylose column. The unbound fraction that contained the chemokine was concentrated and run over a Superdex 200 gel filtration column. In all cases, the proteins eluted in the void volume as a unique peak corresponding to aggregates (data not shown). Chemokines produced through this MBP fusion approach were therefore not further characterized.

### Insoluble Expression and Refolding

Expression was then attempted *via* the approach most commonly used for chemokine production: expression in *E. coli* under an insoluble form in inclusion bodies [2]. For that purpose, the synthetic genes encoding CCL5 and CXCL12 mentioned above were cloned into the pET-20b expression vector. Once again, various constructs of each chemokines were designed for various purposes. CXCL12 was C-terminally appended with either a hexahistidine tag or a Strep-tag. The two constructs were declined under two versions - with or without an LT Tag - resulting in 4 constructs: CXCL12-HIS, CXCL12-LT-HIS, CXCL12-Strep, CXCL12-LT-Strep. For CCL5, solely 3 constructs were created, CCL5-Strep, CCL5-LT-Strep and CCL5 devoid of any tag. Those seven versions of chemokines are schematically represented in Figure 2B.

Protein expression was induced in BL21(DE3) cells. Solubilization of inclusion bodies and the subsequent purification steps completely differed between the LT tag containing constructs and the others. That is mainly explained by the great pI discrepancy between those two sets of proteins, the formers display an acidic pI (below 6.5) while the latter are highly basics with pI values above 9 (see Figure 2B). LT tag bearing proteins were thus solubilized in a 6 M Urea buffer, while the others were treated with 7.5 M Guanidine buffer. Solubilized inclusion bodies were loaded on a SDS-PAGE gel and the amount of overexpressed proteins was roughly appreciated (Figure 2C). One clear observation from this analysis was the clear increase of expression triggered by the LT tag addition. Indeed, for all constructs, the LT tag containing one was far better expressed (CXCL12-LT-HIS *vs.* CXCL12 -HIS, CXCL12-LT-Strep *vs.* CXCL12 -HIS, CCL5-LT-Strep *vs.* CCL5-Strep).

**Figure 3:**
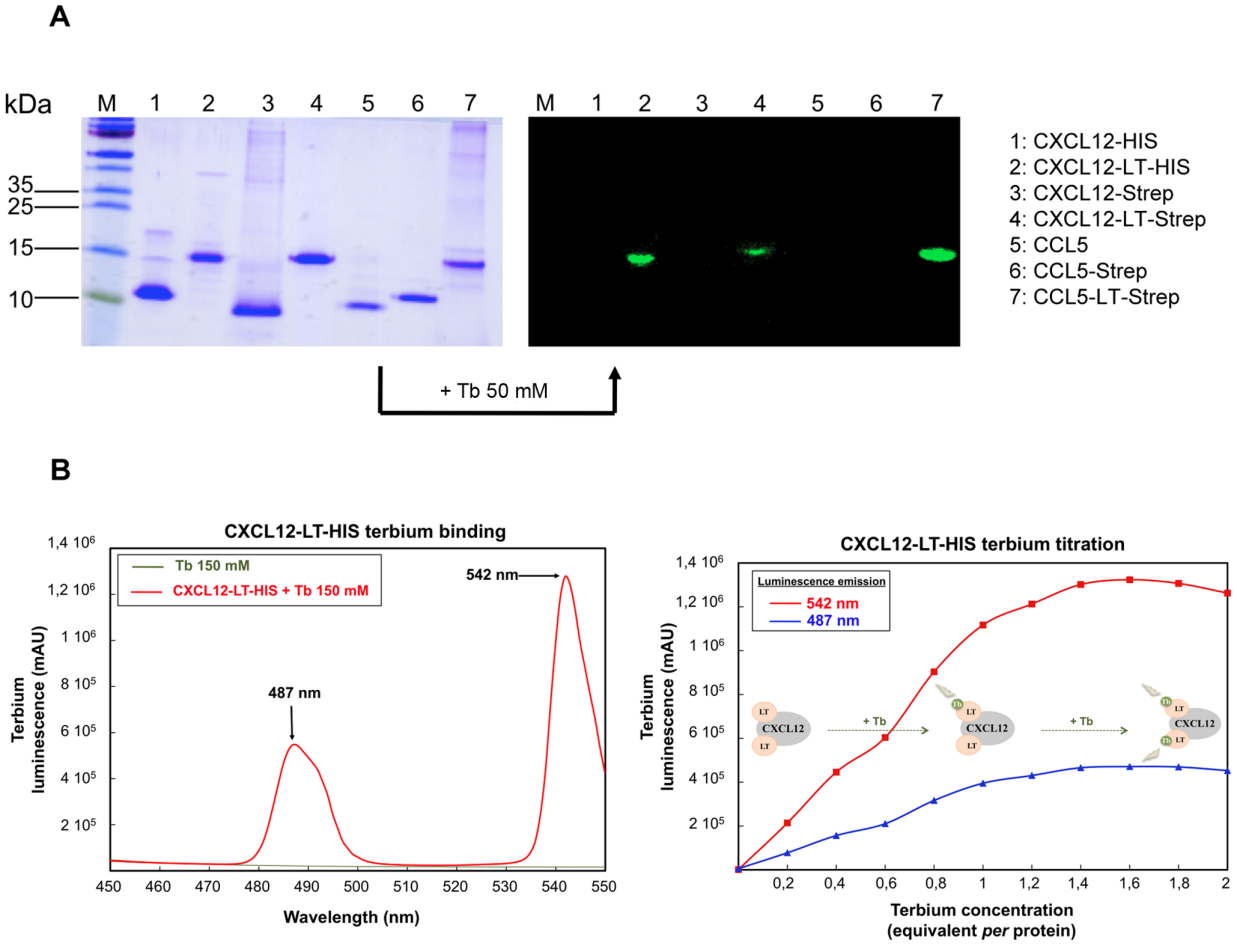
SDS-PAGE analysis of purified chemokines and CXCL12-LT-HIS terbium titration. A. SDS-PAGE analysis of the purified recombinant chemokines. Proteins were loaded onto a 15% SDS-PAGE polyacrylamide gel in denaturing buffer, subjected to electrophoresis at 220 V for 1 hour and stained with a Coomassie staining solution or treated with 50 µM of TbCl3. Luminescent bands associated with the chemokine-LT constructs were visualized on a UV-transilluminator with contrast enhancement. M: Protein mass ladder. B. Tryptophan-sensitized luminescence spectra of a terbium solution with (in red) or without (in green) the purified CXCL12-LT-HIS protein (75 µM in 20 mM Hepes, pH 8.0; 100 mM NaCl) were recorded between 450 and 550 nm (left panel) as indicated in the materials and methods section. As illustrated, significant luminescence amplification is observed at 487 and 542 nm (black arrows) when the terbium ion is trapped by the lanthanoid-binding tag engineered on the purified recombinant chemokine. The CXCL12-LT-HIS terbium titration (right panel) is performed with the same purified CXCL12-LT-HIS protein sample (75 µM) using increasing terbium concentration (15 to 150 µM). After each addition, the solution was mixed and the luminescence emission spectrum was recorded between 450 and 550 nm. For each spectrum, the absorbance values at 487 (blue curve) and 542 nm (red curve) were selected and the CXCL12-LT-HIS terbium binding was visualized following the increase of the luminescence emission versus terbium concentration (expressed in equivalent per protein).

Refolding of the LT tag devoid chemokines was achieved by a 7.5 fold rapid dilution while LT tag chemokines were refolded by drop-wise dilution into a large volume of refolding solution. Amazing discrepancies were observed during the refolding. Comparison of the same constructs with or without LT tag showed that while most of the protein remained soluble in LT tag containing constructs, apart from CXCL12-HIS which refolded very well, all the proteins deprived of LT tag precipitated. Another noticeable observation was the penalty triggered by the Strep-tag. Indeed, comparison of CXCL12-HIS and CXCL12-Strep constructs showed that the presence of the Strep-tag led to the precipitation of a significant amount of protein along the refolding process. After refolding, a first step of purification over an ion-exchange chromatography was performed. The basic non-LT tag containing chemokines, were retained by a Capto S column, while their LT counterparts, with acidic pI were purified over a Q-Sepharose column. After concentration, the proteins were further purified over a Superdex 200 gel filtration. CXCL12 proteins eluted as a nice single monomer peak, while CCL5, which is well known to oligomerize, eluted as higher molecular weight complexes (data non shown). The structural integrity of the samples had to be evaluated. First of all, ion-spray mass spectrometry demonstrated that the oxidized proteins had four mass units less than the reduced forms due to the formation of the chemokine characteristic two-disulfide bridges (Figure S1 shows CXCL12-HIS and CXCL12-LT-HIS analyses). Besides, circular dischroism spectra were performed on two of the produced chemokines, CXCL12-HIS and CXCL12-LT-HIS (Figure S2). They were similar to what was previously described for untagged CXCL12 [16] and therefore confirm the innocuousness of added tag for the refolding of the chemokines. The purity of the prepared protein can be appreciated on Figure 3A (left panel).

The enhancement of the fluorescence triggered by addition of terbium to the CXCL12-LT-HIS strengthens the assumption of a correct folding of the protein (Figure 3B, left panel). Besides, terbium titration demonstrated that a 1.5∶1 molar ratio of Tb:protein was necessary to saturate the sites suggesting than more than one lanthanoid site is active (Figure 3B, right panel). The fluorescent properties of the LT tag are clearly illustrated on Figure 3A (right panel) that displays the visualization of the fluorescent chemokines on a SDS-PAGE gel that was simply bathed into a terbium containing solution.

### Qualitative and Quantitative Functional Characterization of Tagged Chemokines

**Figure 4:**
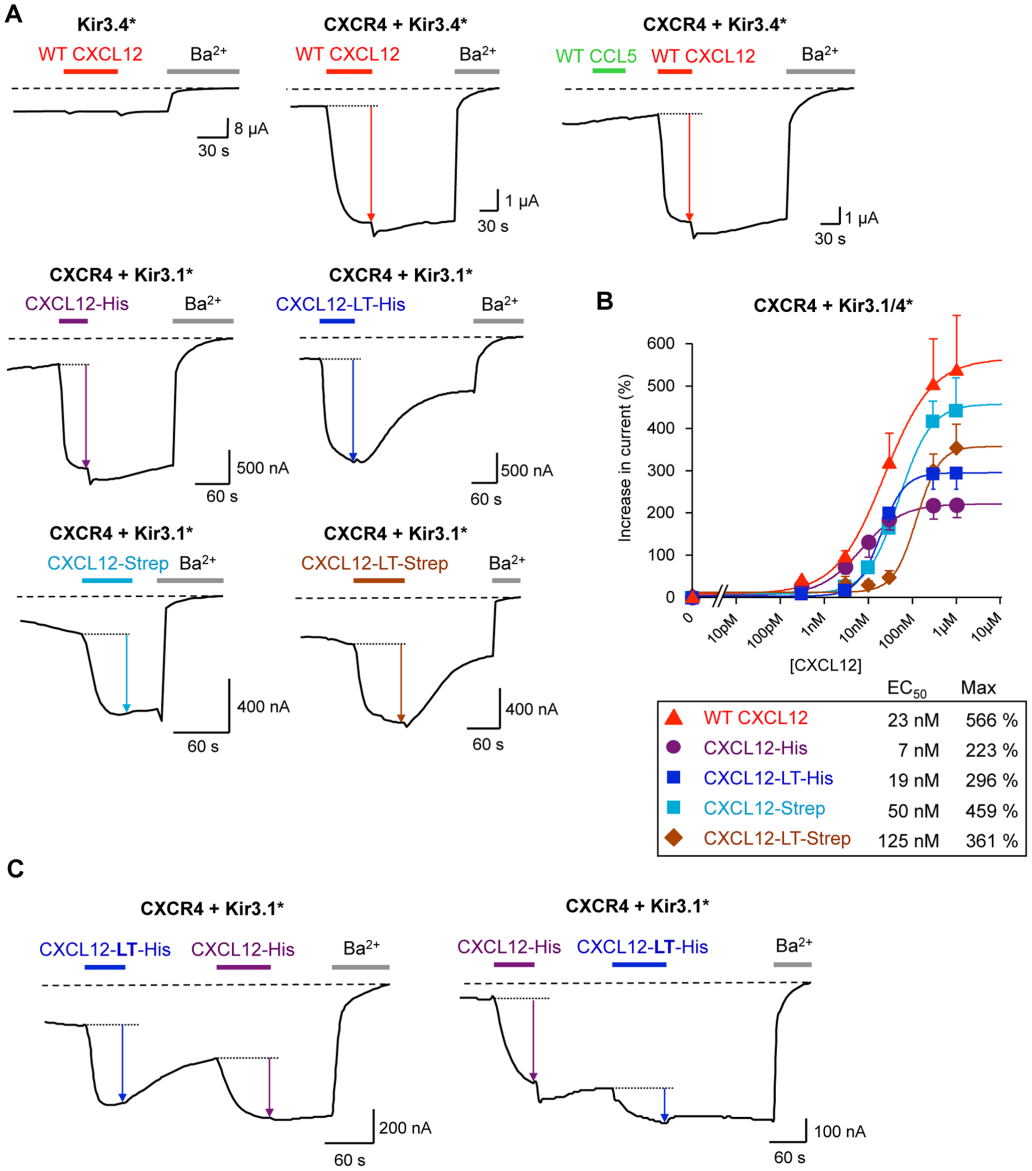
Functional characterization of tagged-CXCL12 chemokines. A. Representative TEVC recordings performed on *Xenopus* oocytes co-expressing CXCR4 and Kir3.1* or Kir3.4*. Different oocytes are used for each recording. The colored arrows represent the channel activation induced by chemokine-binding on CXCR4 and subsequent G proteins activation. The chemokines concentration is 1 µM while the Barium concentration is 3 mM. B. Dose-response curves of the indicated CXCL12 chemokines. Chemokine concentrations are applied gradually on the same oocyte and each point of the curves is the mean +/− s.e.m. of different recordings from different oocytes. C. Sequential application of CXCL12-HIS +/− LT tag during the same recording on the same oocyte but in different order shows the rapid “reversibility” of the activation induced by the LT-tagged CXCL12 chemokine. Each point is an average of 4 to 13 recordings.

Tagged and purified CXCL12 chemokines at 1 µM was continuously flowed over *Xenopus* oocytes expressing CXCR4 and Kir3 channels (Figure 4A). This concentration is 1000 times higher than the reported EC_50_ (1.2 nM) [17] and ensures to be in saturating conditions to trigger a maximal response as shown in Figure 4B. Figure 4A reveals that, independently of the added tag, all modified CXCL12 chemokines are able to activate Kir3.1* or Kir3.4* channels, indicating their ability to bind and activate CXCR4. The negative controls (Kir3.4* alone and CCL5, a CXCR4 non binding chemokines) confirm the specificity of action of the CXCL12 chemokines on the receptor.

As all modified and purified chemokines seemed functional, we performed dose-response experiments by subsequently applying increasing concentrations of chemokines onto the same oocyte. Several oocytes expressing the same constructs are tested with the same protocol and the chemokine-evoked change in current is normalized and averaged to obtain the curves in Figure 4B. These dose-response curves show differential effects induced by the tags depending on their nature and their combination.

For CXCL12, insertion of the HIS-tag significantly decreases by a factor of 2.5 its efficacy (maximal effect) while its apparent affinity (EC_50_) remains in the nanomolar range (7 nM *vs.* 23 nM for wt). This result indicates that CXCL12-HIS acts as a partial agonist.

Addition of the LT tags to CXCL12-HIS does not change the partial agonist phenotype with an efficacy 1.9 lower than wt CXCL12 and an EC_50_ (19 nM) similar to the wt chemokine (23 nM).

For CXCL12-Strep, the efficacy remains similar to the wt (1.2 lower than wt) and the apparent affinity is slightly decreased (EC_50_ = 50 nM *vs.* 23 nM), indicating that the Strep tag does not significantly affect the function of CXCL12.

Surprisingly, addition of the LT tags to CXCL12-Strep significantly decreased both the efficacy (1.6 lower than wt) and the apparent affinity (EC_50_ = 125 nM *vs.* 23 nM for the wt). This result implies a partial activation of the receptor and a lower affinity due to the insertion of the LT tag. This combination of the LT and Strep tags appears to be the most deleterious combination for the CXCL12 chemokine activity (lower efficacy, lower affinity).

In order to validate these results, we performed the functional characterization of the same tagged-CXCL12 chemokines with a standard chemotactic assay. Comparison of the results with the Kir3 channel assay is only partially possible due to the limitations of both techniques. The Kir3 assay is suited to the detection and quantification of ligand effects on receptor activation but their physiological effect is only detectable in chemotaxis assays with migrating cells. Conversely, chemotaxis assays are suited to the characterization of chemo-attractive ligands, but they cannot screen non-chemotactic molecules, such as blockers of viral infections. Additionally, the chemotaxis assays are based on the migration of cells in a gradient of ligands, but, at the higher concentrations, the gradient is rapidly dissipated by diffusion, leading to a rapid stop of cell migration. This effect is evidenced by the bell shape of the dose-response curves. Consequently, the maximal effect is reached before saturation of the receptors, yielding different EC_50_’s for the chemotaxis tests and the receptor activation tests. Moreover, the activity of low-affinity ligands becomes barely detectable in chemotaxis assays due to the requirement for high concentrations of those ligands.

**Figure 5:**
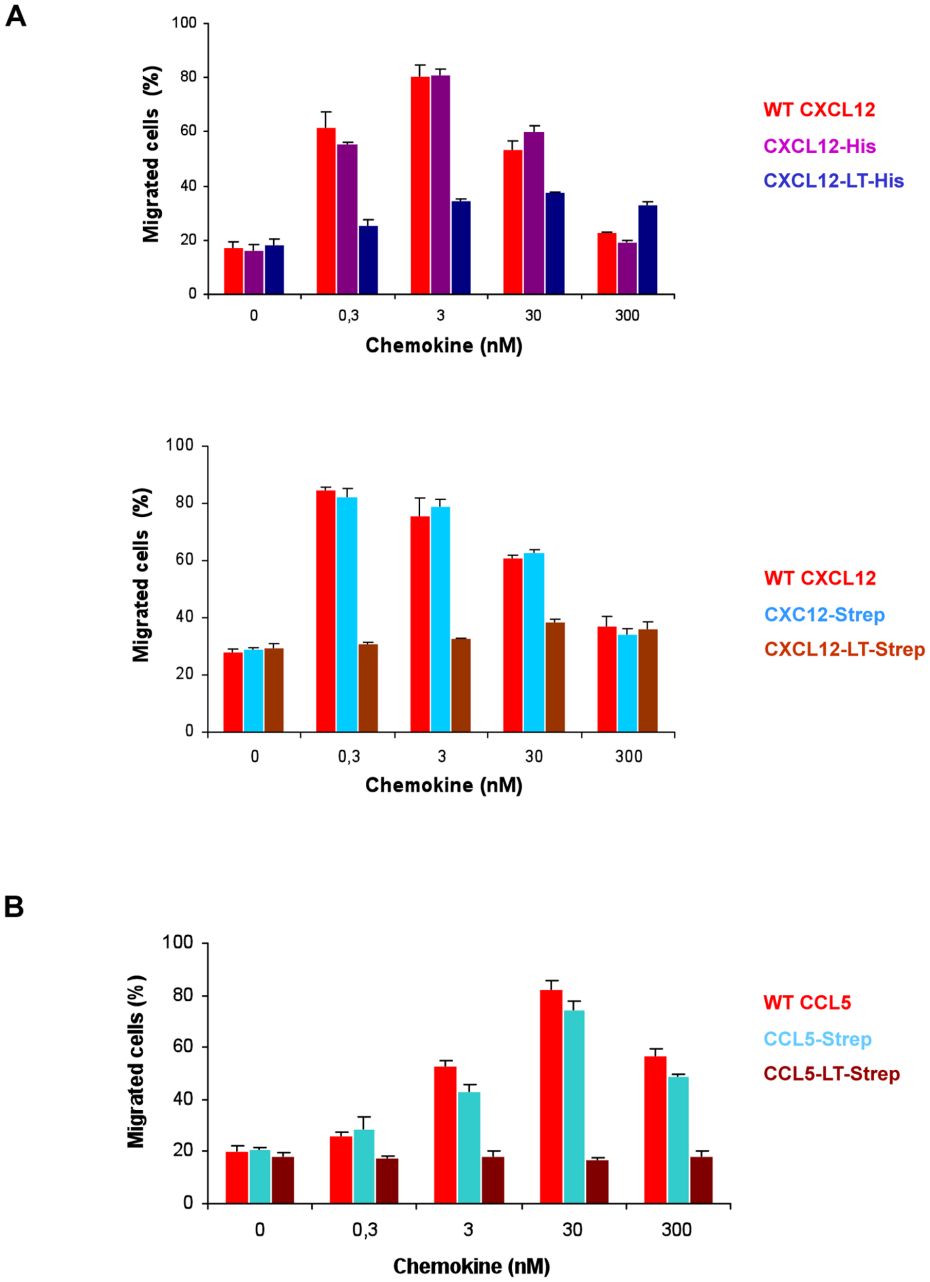
Control of the activities of purified and refolded chemokines by a standard chemotaxis assay. Dose-dependent chemokine-induced migration of Jurkat cells was evaluated using a transwell system. Varying concentrations (0 to 300 nM) of WT CXCL12 or tagged-CXCL12 (A) and WT CCL5 or tagged-CCL5 (B) were added to the lower chamber. The results are expressed as percentage of input cells that migrated to the lower chamber over 3 hours from 3 replicate wells per condition (data are means ± SD). For CCL5-LT-Strep averaged values are slightly lower than the control at 0 nM clearly indicating an absence of chemotactic activity induced by this chemokine.

The dose-response results of chemotaxis assays on the tagged CXCL12 chemokines are presented in Figure 5A. All CXCL12 chemokines induced chemotaxis. Figure S3 shows more clearly the weak response to the LT-tagged chemokines. Surprisingly, while HIS and LT-HIS tagged CXCL12 have similar properties in the Kir3 channel assay, the CXCL12-LT-HIS activity is drastically decreased in the chemotaxis assay. For the Strep-tagged CXCL12 chemokines, the results are more consistent as CXCL12-Strep shows a response similar to the wt CXCL12 in both assays, and introduction of the LT tags induces a decrease of efficacy and apparent affinity in both assays. However, this effect is more pronounced in the chemotaxis results than in the Kir3 assay results. The different results obtained with the two assays suggest that some parameters required for chemotaxis of mammalian cells is not detected by the Kir3 channel reporter in *Xenopus* oocytes.

Thus, the Kir3 channel assay is an approach complementary to the cell-based assays and permits a quantitative assessment of the interactions of chemokines with their receptors.

Another advantage of the Kir3 assay is the real-time recording of chemokine effects. Thus, the insertion of the LT-tags modified the de-activation kinetic of CXCR4. The time of de-activation is observed during the washing step of the oocyte after the chemokine application. For instance, application of the wt CXCL12 on CXCR4+ Kir3.4* (Figure 4A upper right trace) shows a very slow de-activation of the receptor during the ligand washing. Insertion of the HIS- and Strep-tag (Figure 4A, the 2 lower left traces) does not change this kinetic. Surprisingly, insertion of the LT-tags in CXCL12-HIS and CXCL12-Strep engenders a relatively rapid de-activation of CXCR4 in less than 4 min (Figure 4A, the 2 lower right traces). Sequential applications of non-LT-tagged and LT-tagged CXCL12 in different orders confirm this kinetic effect induced by the LT-tags, as shown in Figure 4C.

This change of de-activation time is difficult to interpret as several parameters could be involved (binding and dissociation kinetics, interaction with other membrane components such as glycosaminoglycans…). The simplest hypothesis is an increase of the dissociation kinetic of the LT-tagged CXCL12, especially in the k_off_ rate.

**Figure 6:**
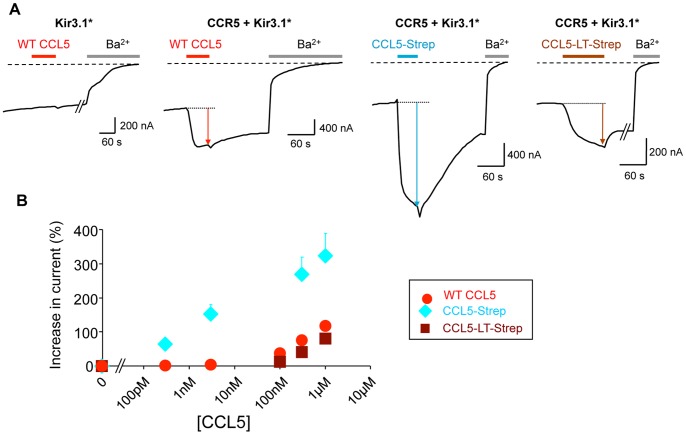
Functional characterization of the tagged-CCL5 chemokines. A. Representative TEVC recordings performed on *Xenopus* oocytes expressing Kir3.1* or co-expressing CCR5 and Kir3.1* and subjected to applications of the indicated CCL5 chemokines at 1 µM. B. Dose-response curves of the tagged and non-tagged CCL5 chemokines. Due to the low concentration of the mother solutions, the highest concentration cannot be extended. Each point is an average of 3 to 25 recordings.

Similar characterizations of CCL5-tagged chemokines were performed on CCR5+ Kir3.1* expressing oocytes. Figure 6 shows that all purified chemokines are active and the negative control with Kir3.1* alone confirms the specificity of receptor-mediated activation of the channel. However addition of Strep and LT-Strep tags also modifies the functional properties of this chemokine but on different parameters than for CXCL12.

Thus, insertion of the Strep-tag induces, in the range of the tested concentrations, a greater efficacy and a higher apparent affinity than the wt CCL5 (Figure 6B). Addition of the LT tags in CCL5-Strep abolishes the Strep-tag effects showing a similar dose-response curve as the wt CCL5. Due to the low apparent affinity of CCR5 for the CCL5 chemokines and the available concentration of the purified chemokine stocks (micromolar range), we were not able to reach the saturation plateau in our conditions. Consequently, reliable fittings were not possible.

Comparison with the chemotaxis results (Fig. 5B) demonstrated once more dissimilarities in the effect of the tags on chemokine activities. The Strep-tagged CCL5 showed a chemotaxis profile comparable to that of the wt chemokine, while it displayed a higher efficacy in the Kir3 assay. Conversely, introduction of the LT tag is deleterious for the chemotaxis activity of CCL5, but its activity on the receptor is unchanged compare to the wt chemokine in the Kir3 assay. These differences of assay-dependent chemokine responses illustrate the importance of complementary assays for the functional characterization of chemokines, both at the molecular level of the receptor and at the cellular level.

Concerning the time of de-activation, the Figure 6A shows a slow de-activation of CCR5 after application of the wt CCL5, which was expected based on the previous CXCL12 results. Surprisingly, insertion of the Strep-tag in CCL5 induces a rapid de-activation. Moreover, addition of the LT tags abolishes this effect and displays a slow de-activation similar to the wt. Those effects of Strep and LT tags on the time of de-activation are the exact opposite of the effects observed for CXCL12.

## Conclusion

CXCL12 and CCL5 chemokines are essential not only for their physiological role in lymphocytes B chemotaxis, but also in their purified forms for the molecular characterization and biochemical studies of their related receptors involved in HIV entry. The pecuniary value of the chemokines reduces their affordable quantity and drastically limits their use for long-standing and structural studies of chemokine receptors. To overcome this obstacle, one solution is to express and purify those ligands using a bacterial expression system. Classical tags such as polyhistidines sequences, maltose binding proteins (MBP) or Strep-tags were added to the proteins of interest to facilitate their purification by affinity chromatography. Lanthanoid binding tags were appended for specific applications such as fluorescence imaging of spectrometry. The tracking of chemokines or receptor/chemokine complexes *via* the fluorescent properties offered by the LT tags is clearly supported by the recent advances made on time-resolved FRET approaches based on tagged ligands or GPCRs with lanthanoid cryptates for functional studies in cell [18] and *in vivo* [19].

We describe here the effects of such modifications of CXCL12 and CCL5 chemokines in all steps of their preparation process from their expression in bacterial inclusion bodies to the receptor activation. We demonstrated that tags such as MBP are proscribed for efficient production of both chemokines while the lanthanoid binding tags increased their expression level and the refolding efficacy.

Using a convenient functional assay based on Kir3 channels activation correlated to chemokine receptors activation in *Xenopus* oocytes, we highlighted the selective and unpredictable effects of tags on the chemokine function. The major strength of the technique is that without having to struggle with membrane protein expression, we had an easy access to experiments that could be delicate with other approaches such as the determination of an EC_50_ by dose response tests or competition assays. Besides, it seems that internalization processes are rather limited in *Xenopus* oocytes [20], which clearly ease ligand binding assays as it enables the possibility of successive perfusions. Single or multiple tags induce selective effects depending on their nature, their combination and the chemokines. Consequently any modifications of chemokines, even at their C-terminus, can generate significant alterations of their properties and a complete functional characterization is a prerequisite before their utilization.

This report provides functional details of CXCL12 and CCL5 chemokines fused to some of the most common affinity tags and guides the selection of the most appropriate tags for specific studies of the CXCR4 and CCR5 receptors. The same approach could be largely used and adapted in the development and optimization of drugs targeted towards those two GPCRs.

## Supporting Information

Figure S1 **CXCL12-HIS and CXCL12-LT-HIS ion-spray mass spectrometry analyzes.** Protein samples (50 µL at 1 mg/mL) were diluted in 50 µL formic acid 0,1%, desalted on line using a HPLC-connected MacroTrap column (Michrom) and eluted with 70% of solvent (95% acetonitrile, 5% water, 0,03% formic acid). Protein masses were determined using an electrospray TOF mass spectrometer (Agilent, LC/MSD TOF) directly coupled with the HPLC system (Agilent 1100 series).
(TIF)

Figure S2 **CD spectra of 20 mM CXCL12-HIS (blue diamond) and CXCL12-LT-HIS (red square) were measured in sodium phosphate buffer at pH 6,0 and 8,0, respectively.** Molar ellipticity is reported as [q] × 10^3^ degree/cm^2^/dmol.
(TIF)

Figure S3 **Chemotactic activities of CXCL12-LT-His and CXCL12-LT-Strep chemokines.** Jurkat cells were allowed to migrate for 3 h using a transwell system. Various concentrations (0 to 300 nM) of CXCL12-LT-His or CXCL12-LT-Strep were added to the lower chamber. The results are expressed as percentage of input cells that migrated in the lower chamber (data are means ± SD). *, *p*<0.01 compared with the control without chemokine.
(TIF)
