# Supplementary figures and images for: C-Terminal Engineering of CXCL12 and CCL5 Chemokines: Functional Characterization by Electrophysiological Recordings
Source: PLoS One. 2014 Jan 31;9(1):e87394. doi: 10.1371/journal.pone.0087394 (PMC3909184; doi:10.1371/journal.pone.0087394)

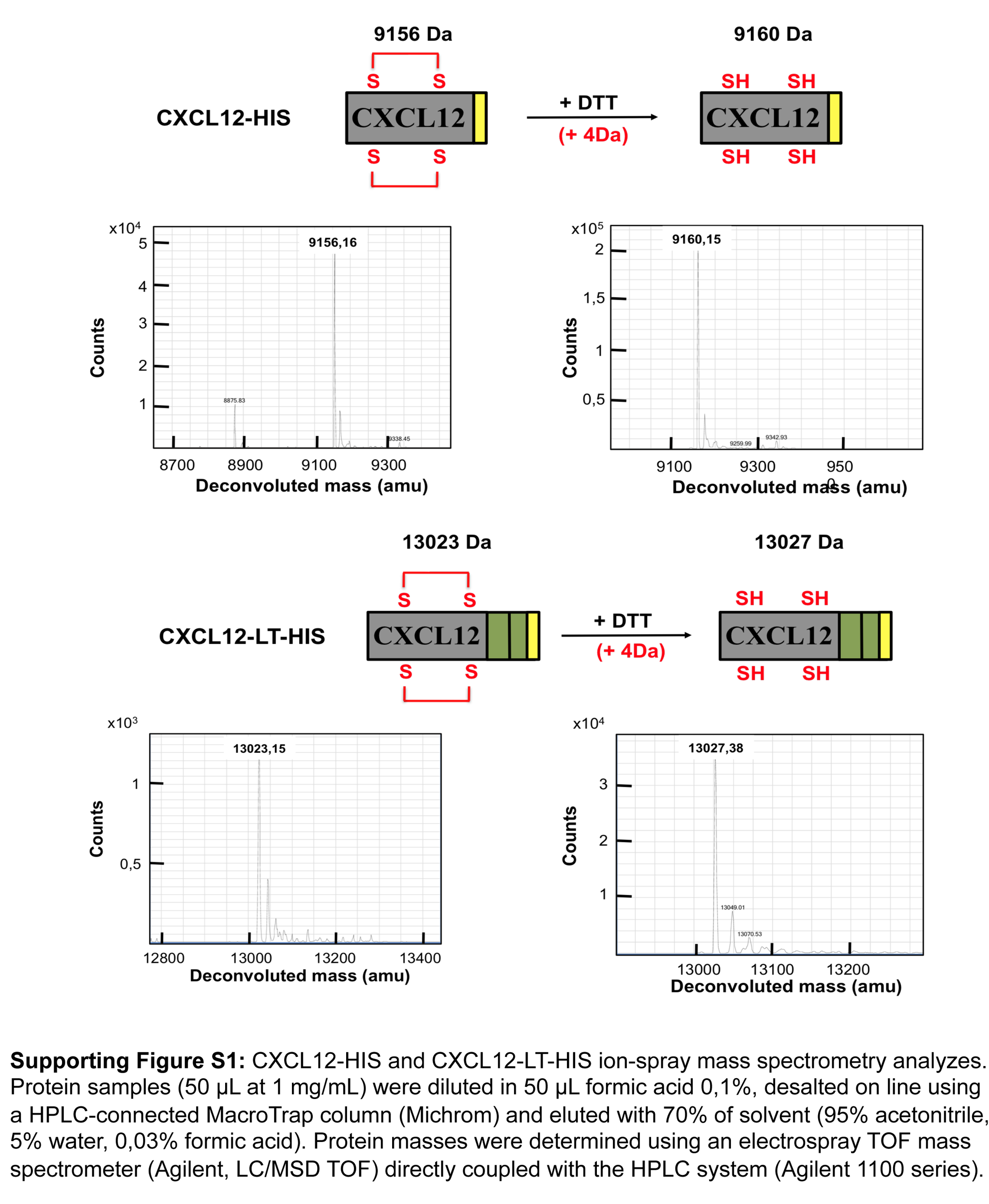

Supplement: Figure S1 — CXCL12-HIS and CXCL12-LT-HIS ion-spray mass spectrometry analyzes. Protein samples (50 µL at 1 mg/mL) were diluted in 50 µL formic acid 0,1%, desalted on line using a HPLC-connected MacroTrap column (Michrom) and eluted with 70% of solvent (95% acetonitrile, 5% water, 0,03% formic acid). Protein masses were determined using an electrospray TOF mass spectrometer (Agilent, LC/MSD TOF) directly coupled with the HPLC system (Agilent 1100 series). (TIF) [file pone.0087394.s001.tif]

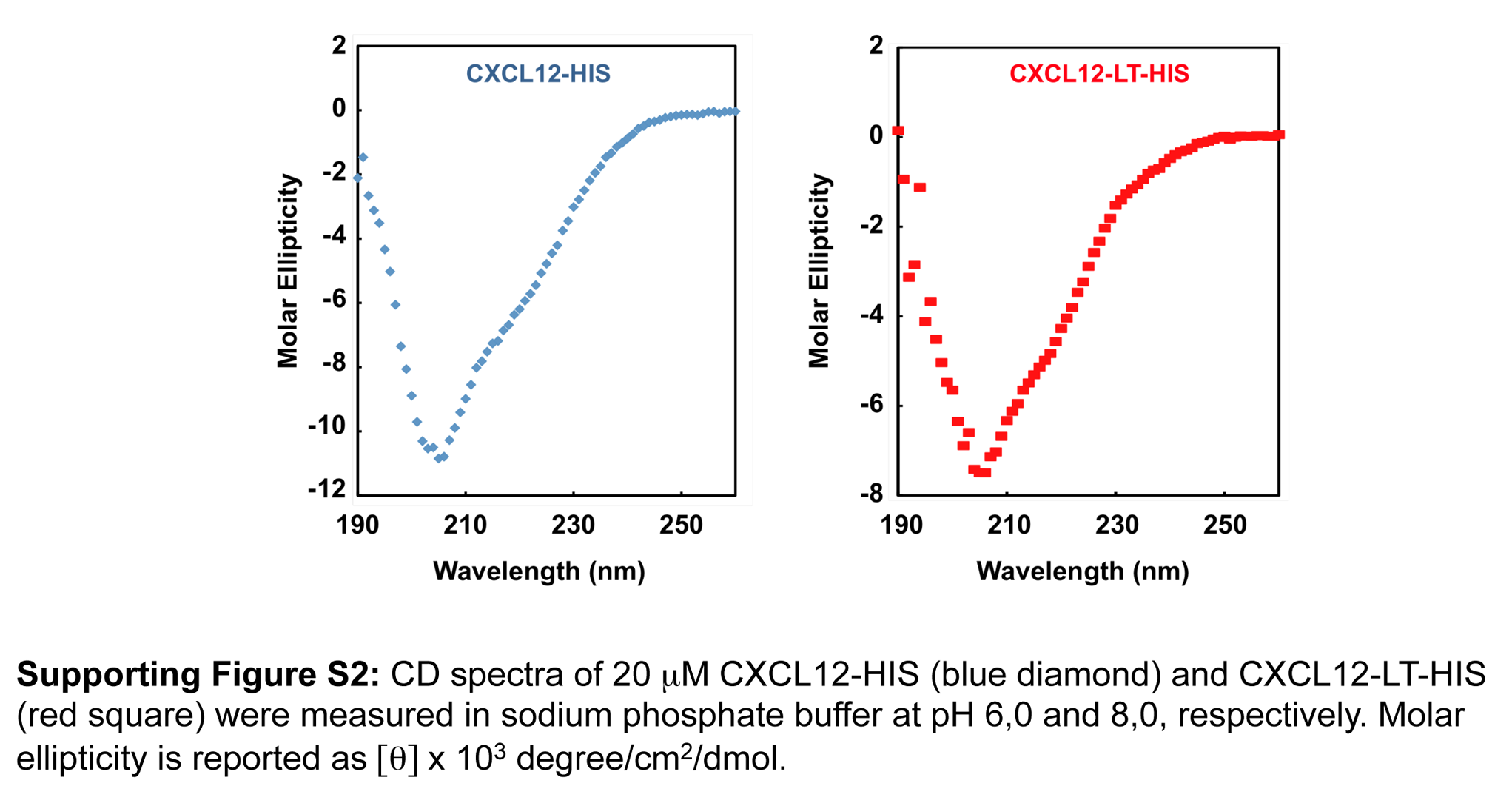

Supplement: Figure S2 — CD spectra of 20 mM CXCL12-HIS (blue diamond) and CXCL12-LT-HIS (red square) were measured in sodium phosphate buffer at pH 6,0 and 8,0, respectively. Molar ellipticity is reported as [q] × 103 degree/cm2/dmol. (TIF) [file pone.0087394.s002.tif]

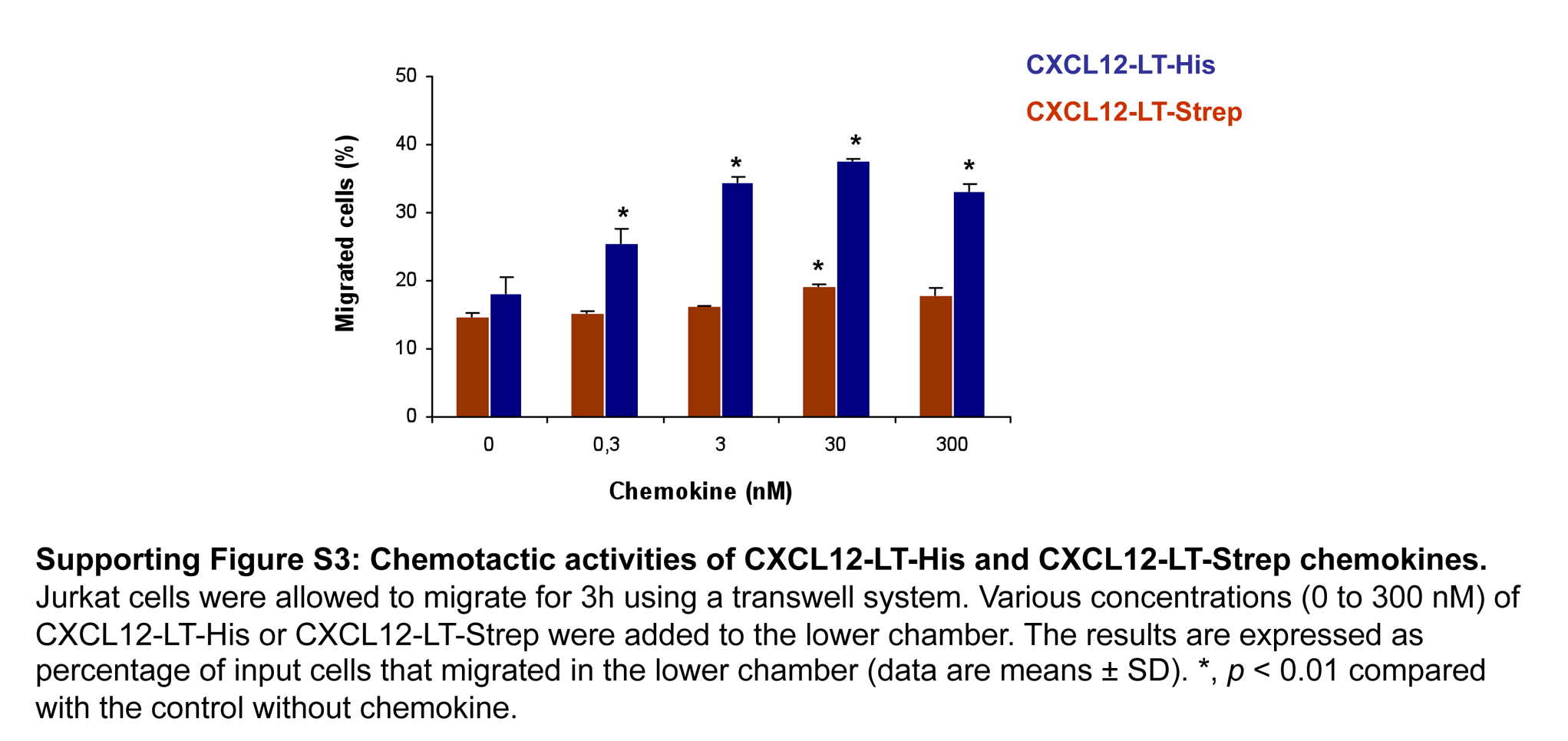

Supplement: Figure S3 — Chemotactic activities of CXCL12-LT-His and CXCL12-LT-Strep chemokines. Jurkat cells were allowed to migrate for 3 h using a transwell system. Various concentrations (0 to 300 nM) of CXCL12-LT-His or CXCL12-LT-Strep were added to the lower chamber. The results are expressed as percentage of input cells that migrated in the lower chamber (data are means ± SD). *, p<0.01 compared with the control without chemokine. (TIF) [file pone.0087394.s003.tif]
